# An Exploration into the Determinants of Noncommunicable Diseases Among Rural-to-Urban Migrants in Periurban South Africa

**Published:** 2010-10-15

**Authors:** Ruth Stern, Thandi Puoane, Lungiswa Tsolekile

**Affiliations:** University of the Western Cape; University of the Western Cape, Bellville, South Africa; University of the Western Cape, Bellville, South Africa

## Abstract

**Introduction:**

Noncommunicable diseases are increasing in developing countries, exacerbated by growing urbanization. We examined the experiences and perceptions about noncommunicable diseases of people who migrated from rural areas to urban Cape Town, South Africa.

**Methods:**

We conducted a qualitative study in an impoverished periurban township that has a noncommunicable disease prevention program, including health clubs. We used in-depth interviews, participatory reflection and action groups, and focus group discussions.

**Results:**

Participants described changes in eating patterns and levels of physical activity. These changes were a result of socioeconomic and environmental constraints. However, respondents were not concerned about these changes. Despite hardships, they were pleased with their urban lifestyle. Furthermore, they approved of their weight gain because it signified dignity and respect. Participants who attended health clubs found them informative and socially and emotionally supportive.

**Conclusion:**

The study highlighted the complexity of the risk factors for noncommunicable diseases and the need to develop prevention strategies that extend beyond the traditional focus on diet and exercise.

## Introduction

The prevalence of noncommunicable diseases, previously associated with developed countries and more affluent populations, is increasing in poorer countries (1,2). Recent data show that of the 10 countries where the rates of diabetes are highest, 7 are developing countries. The death rate from diabetes is 4 times higher in sub-Saharan Africa than the world average (3).

The growing trend has been exacerbated by the rapid increase in urbanization (4), which includes migration of impoverished people from rural areas (5). This migration contributes to the increasing inequities between the rich and poor (6); people who live in poverty tend not to benefit from the higher living standards of urban life (7,8). The inequity is further reinforced by an unequal distribution of resources because the scale of urbanization exceeds the capacity of governments to cater for the multiple needs of a population living in poverty (6,9).

A known consequence of urbanization is obesity, a major risk factor for noncommunicable diseases (10). Several contributing factors have been cited, including the limited availability of affordable, healthy food in poorer areas, combined with the increased availability of fast foods that are high in fats and sugar (11). As communities become more urbanized, physical activity declines because of sedentary employment (12), limited outdoor space (13,14), and high rates of street violence (15). Cultural beliefs and practices also contribute: obesity is valued in many African cultures because it is associated with dignity, wealth, and being treated well by one's husband (16), and weight loss is regarded as a source of stigma and a sign of disease, in particular HIV/AIDS (17). Despite awareness of these different factors, the focus on noncommunicable disease prevention remains on lifestyle change (18). This focus applies to both developed and developing countries, and it includes the South African national and provincial governments (19,20).

Freudenberg (18), a strong advocate of a broad, social determinants-based approach to noncommunicable disease prevention, has challenged universities to influence the direction of noncommunicable disease prevention. Consequently, we explored the experiences and perceptions of members of a migrating community to improve our understanding of the factors that affect noncommunicable diseases.

## Methods

### Study setting

The study was undertaken in Khayelitsha, a periurban settlement in Cape Town, which has grown substantially because of recent rural-to-urban migration (7). Population estimates vary from 330,000 to 1 million (21,22). Most residents live in poverty: 55% live below the poverty line compared with the Cape Town average of 25% (21), and although some find employment, 41% are economically inactive (21), surviving on the marginal informal economy (8).

The School of Public Health at the University of the Western Cape initiated the Khayelitsha noncommunicable disease prevention initiative in 2000 after being approached by community health workers who were concerned about the levels of diabetes and hypertension in their community. The first task was to work with the community health workers to enhance their understanding of the risk factors and determinants of noncommunicable diseases. The community health workers then led a prevention intervention that included public information events, group *fun walks* that facilitated safe outdoor activities, and screening for high blood pressure and diabetes. In 2005, weekly health clubs were established to broaden the community focus (13,23). The initial focus of the clubs was lifestyle change through discussions, cooking demonstrations, and exercise sessions. The aim, however, was to expand the focus to take account of the broader determinants of noncommunicable diseases. To achieve this, a clearer understanding of the community's circumstances was deemed necessary.

### Data collection

Data were collected in 2 phases — a quantitative study described elsewhere (22) and a qualitative study, which is the focus of this article. The qualitative study was necessary to understand the respondents' perceptions about the circumstances and effect of their migration. We used 3 approaches to enhance the data collection and allow for triangulation. These approaches were in-depth interviews (conducted in February and March 2007), participatory action and reflection groups (described below, conducted in June 2007), and focus group discussions (conducted in September 2007). All components of the qualitative study took place in the community.

We used purposive sampling to identify 55 people (45 women and 10 men for the study). (Women were not deliberately oversampled.) Each respondent participated in only 1 activity, and no one declined to take part. We selected 10 respondents from the quantitative study for the interviews (3 of whom were club members). The community health workers selected the remaining respondents from their programs. The only selection criteria were that all respondents had to have lived in Khayelitsha for 5 years or less, and some respondents had to be club members. Given this sampling procedure, some respondents would have had some knowledge of noncommunicable disease prevention.

One bilingual author led all 3 approaches so that she could build on the data and probe for clarification as required. The local language, isiXhosa, was used throughout. We used in-depth interviews to gain an initial understanding, focusing on changes in diet and physical activity, what helped participants cope, and the barriers they faced. The interviews averaged 45 minutes to 1 hour.

We then conducted 2 participatory action and reflection groups: 1 of club members (10 people) and 1 of nonmembers (15 people). Because the focus of the researcher was on facilitating the groups, we created a more dynamic, respondent-led environment. These groups lasted from 1.5 to 2 hours and consisted of 3 activities: Venn diagrams, which explored social interactions and sources of information; ranking and sorting, which discussed coping mechanisms; and problem-tree analyses, which probed for reasons for weight gain. Finally, we led two 45-minute focus group discussions, 1 of club members and 1 of nonmembers (10 people in each). These discussions maximized respondent interaction, focusing on social integration, challenges, and reasons for weight gain. The interviews and focus groups were recorded for accuracy, transcribed, and translated by 2 people independently. A researcher checked the accuracy of the discussions with the respondents at the end of each session. We identified themes and priorities through content analysis of the transcripts and the notes and charts from the action and reflection groups. No statistical software was used. All respondents consented to take part and knew they could withdraw without repercussion. We obtained ethical clearance from the University of the Western Cape.

## Results

### Reasons for leaving the rural areas

Overwhelmingly, people left a life of poverty and hard work to come to Khayelitsha. Most were farm workers, and women — who made up most respondents — also fetched water, carried firewood, and attended to their homes and families. Job security was a major concern because options were limited beyond the agricultural sector.

We would work from morning to sunrise and right throughout the day . . . We had to do this as this was our way of living.

Access to health services was limited, and transportation was difficult.

In the rural areas hospitals are far, and when you fall sick, suddenly you struggle to get transport and end up not going.

### Perceptions and reality of urban life

Part of the lure of the city was the perception of an easier and more comfortable lifestyle, and for those who found employment, circumstances improved. Typically, however, circumstances remained difficult.

Not getting a job was one of my biggest challenges as I'm still fit to work. If I can get a job, I think things will be better.

We did not have a place of our own; we did not even have a bed. We used to lay cardboards and we would sleep on them.

Furthermore, participants were concerned about personal safety, which limited how much people were willing to venture beyond their homes. Social integration was also more difficult than they had anticipated. Several respondents described being lonely and isolated on arrival and missing friends and family.

When I needed something [before], I knew who to approach as I already had a relationship with those people. Here I cannot ask as I don't know many people.

### A sense of fulfillment and opportunity

Despite these concerns, most respondents were pleased to be in the city. This seemingly contradictory view related to the relative ease of their lives and their growing integration into a new community.

Initial contacts with family expanded to include new friendships and broader social networks. Neighbors — an accessible source of local knowledge — were particularly valuable. Respondents also learned about formal institutions such as health services and the church, which provided support and information.

When you belong to a church, there is constantly an umbrella that protects you and comforts you. You tend to accept what has happened even though it may be difficult.

The respondents were learning to negotiate a new environment with opportunities that did not exist in rural areas. They were becoming part of an urban society that, while difficult, provided diversity and the potential for personal development. These achievements created a sense of well-being and satisfaction.

My life [before] was centered on my household and the only thing I thought about was to go to the field. I did not think of anything further than that. When I arrived here, I started to think more about things.

My life has changed since moving to the city because I feel like a different person.

### Effects on diet, exercise, and weight

The adoption of an urban lifestyle affected the diet and physical activity of the respondents. They ate more often and spent less time preparing food. However, the food they ate was often high in sugar and fat. Food choices were limited by budget and the poor range of food available locally. The exposure to, and desire for, a more urban diet, including fast food, was often perceived as progress from rural diets. Food could also be bought on credit from local street vendors.

Here you are always surrounded by food . . . You can even eat 4 times a day.

Physical activity, part of daily life in the rural areas, was now reduced, because of a combination of reality and because of their current circumstances. Although physical activity was considered to be a requirement or disadvantage of their past life, respondents no longer perceived it as necessary. Basic amenities were more accessible, and for people with jobs, work was less strenuous. The fear of violence during outside activity was also prevalent, particularly among women.

I don't have to fetch water in far-off places. Now I fetch water next to the house.

I have fears as there are too many criminals here.

Walking also became a choice instead of a necessity because other transportation was available.

I do not see the need for walking just for nothing.

Respondents self-reported a substantial amount of weight gain, but weight gain was not a concern. On the contrary, weight gain was seen as symbolic of their success in the city. A loss in weight indicated problems in one's life. A frequent comment was that respondents were overweight because they were happy.

I'm very happy . . . When I'm at home [rural area], they compliment me and say that I'm fat. I tell them that rural life is different from city life.

### The role of the health clubs

Respondents who attended health clubs appreciated the information about risk factors of diabetes and hypertension because they were aware of the increase in health problems in their community.

They teach us how to eat, and they told us that fats are bad as they are the ones causing your heart not to work well. Always using a taxi [a cheap form of transportation] is not right.

But the information was difficult to act on. Apart from practical constraints, family members were an obstacle because they resisted giving up the lifestyle they had become accustomed to. Family members were also tempted by the elevated status of obesity. The discussions in the club helped people address these dilemmas and provided role models of people who had lost weight. The clubs also provided social and emotional support, and the club members described a sense of personal growth and belonging.

In the club, you talk to people and this removes you from your problems. You stop thinking about your problems when you are there.

We share problems and we confide in each other. We've also made friends through the club.

## Discussion

Our findings reinforce previous research that shows how lifestyles change when people from impoverished rural communities migrate to urban areas and gain weight (11-13,23,24). The findings also show how poverty affects the choices that people make (7,14,15).

Respondents described themselves as content with their city lives despite the problems. The food they ate was high in fat and sugar, but it was palatable and readily available. Fast food requires no preparation and can be a status food (25). Respondents were also less physically active than when they lived in rural areas. This was perceived as progress because walking and physical labor in the past represented hardship. The weight gained as a consequence of the urban lifestyle gave respondents a sense of achievement and dignity (16,26), which was reinforced by receiving compliments and respect when they visited their previous homes. These factors resulted in a lack of incentive to change.

This complex situation highlights the challenges for preventing noncommunicable diseases. Focusing only on healthy lifestyles misses the heart of the problem (14). To be effective, strategies must consider the range of social determinants in which choices are made, particularly the cultural perceptions of obesity (10).

The Khayelitsha intervention has adopted a local approach but one that focuses on community development. The health clubs began by encouraging lifestyle changes, using practical measures such as food preparation and exercise classes. However, as demonstrated, their effect has extended further; they provide a supportive environment, build social cohesion, and improve social networks. These effects have facilitated peer education, which further empowers club members. In other words, the clubs develop social capital, which is beneficial for health (27-29). Furthermore, since the study, relationships and networks with relevant nongovernmental organizations (NGOs) and public-sector institutions have strengthened, and new clubs have formed. The conditions in which people live remain a concern, and advocacy for improved facilities and a more supportive environment for noncommunicable disease prevention is now a component of the program. The initial clubs are also role models for other initiatives in Khayelitsha and elsewhere.

Qualitative research has shown the role of socioeconomic and cultural determinants of noncommunicable disease, as perceived by community members (13,23). The complexities of their lifestyles require interventions that look beyond the traditional risk factors of diet and exercise.

Our study has limitations. We used a small sample of people who, because of the sampling procedure, already knew about noncommunicable disease prevention. Whether similar perceptions exist among people with no previous knowledge is not known. Using 3 methods of qualitative research enabled a rich mix of responses. Participatory action and reflection groups were particularly valuable because they empowered respondents and produced interesting data. A more substantive participatory action and reflection study may be beneficial. Finally, the study focused on local circumstances and perceptions about them. A more in-depth study that extended to regional, national, and global aspects would put the data in a wider context.

Despite these limitations, we achieved our objective of demonstrating the need for broad-based interventions to address noncommunicable diseases. The experience of the health clubs also illustrates the value of working with community organizations to strengthen social and community networks to create an understanding of the need for change and for recognizing barriers. Given that increased rural-to-urban migration is part of a global trend, this research shows some of the reasons why noncommunicable diseases are increasingly affecting poor populations and suggests some strategies for addressing the problems. We suggest that strategies shift beyond the individual behavior to address the environment in which people live. Examples include advocacy campaigns to improve access to healthy food and safe outdoor environments for exercise and improved engagement with community-based institutions such as community groups, NGOs, and faith-based organizations to gain access to communities.
